# Breast Cancer in Low- and Middle-Income Countries: An Emerging and Challenging Epidemic

**DOI:** 10.1155/2010/490631

**Published:** 2010-12-15

**Authors:** Arafat Tfayli, Sally Temraz, Rachel Abou Mrad, Ali Shamseddine

**Affiliations:** ^1^Division of Hematology/Oncology, American University of Beirut Medical Center, P.O. Box 11-0236, Riad El-Solh, Beirut 1107 2020, Lebanon; ^2^Department of Internal Medicine, American University of Beirut, Beirut, Lebanon

## Abstract

Breast cancer is a major health care problem that affects more than one million women yearly. While it is traditionally thought of as a disease of the industrialized world, around 45% of breast cancer cases and 55% of breast cancer deaths occur in low and middle income countries. Managing breast cancer in low income countries poses a different set of challenges including access to screening, stage at presentation, adequacy of management and availability of therapeutic interventions. In this paper, we will review the challenges faced in the management of breast cancer in low and middle income countries.

## 1. Introduction

Breast cancer is the most common cancer of women, and its incidence is rising especially in developing countries. Global burden of breast cancer in women, measured by incidence, mortality, and economic costs, is substantial and on the increase. Worldwide, it is estimated that more than one million women are diagnosed with breast cancer every year, and more than 400,000 will die from the disease. 

In low- and middle-income countries (LMCs), the infrastructure and resources for routine screening mammography are often unavailable. In such countries, breast cancer is usually diagnosed at late stages, and, due to inadequate resources, women with breast cancer may receive inadequate treatment or palliative care.

Many barriers are identified for breast cancer patients in LMCs which may correlate with the lower incidence and higher mortality in those countries compared to high-income countries. These barriers include the lack of breast cancer awareness due to poor health awareness and education, lack of screening programs due to the lack of governmental support and inadequate funds, social barriers to early diagnosis and treatment due to low priority for women health issues in predominantly patriarchal developing societies, fear of loss of employment and the social taboo of cancers and misconceptions about cancer treatment and outcomes, lack of standardized treatment protocols with diversity of clinical practice, health care standards and infrastructure, and finally poor followup data and the lack of mortality data.

## 2. Epidemiology

Breast cancer is the most common cancer in women, accounting for 23% of all female cancers around the globe [1]. There is a marked geographical variation in incidence rates, being highest in the developed world and lowest in the developing countries of the third world. However, in recent years, the incidence of breast cancer has shown an alarming increasing trend [2]. It is estimated that 45% of the 1.35 million new cases diagnosed each year, and more than 55% of breast cancer related deaths, occur in low- and middle income countries [3]. An estimated 1.7 million women will be diagnosed with breast cancer in 2020—a 26% increase from current levels, mostly in the developing world [4].

The most widely cited reason for the global increase in breast cancer is the “westernization” of the developing world [2]. Social factors like smoking, alcohol, and obesity are becoming more common in the developing countries and are increasingly accepted [5]. Hormonal risk factors like early menarche, delayed parity, and reduced breast feeding are now being observed in low- and middle-income countries [2]. Potential explanations for this are the wider adoption of the western diet and lower exercise levels.

Breast cancer incidence has, historically, been less common in Asia compared to the West. However, recent statistical data reveal breast cancer to be the leading cause of cancer in Southeast Asian women and second only to gastric cancer in East Asian women and to cervical cancer in women in South-Central Asia. Countries with the most developed registries have documented increases: rates in Japan, Singapore, and Korea have doubled or tripled in the past 40 years, and China's urban registries document 20 to 30% increases in the past decade [2]. In China's commercial center of Shanghai, 55 out of every 100 000 women have breast cancer, a 31% increase since 1997. 

While breast cancer incidence progressively increases with age in the western countries, a different pattern is being observed in Japanese women. The breast cancer incidence rate seems to plateau after the age of 50 in Japanese women [6].

In India, almost 100 000 women are diagnosed with breast cancer every year, with increases concentrated in urban areas, and a rise to 131 000 cases is predicted by 2020 [4]. The incidence of breast cancer has been rising by 0.5% to 2% per year across all regions of India, and in all age groups, but has been even higher in women younger than 45 years of age. Almost half of the Indian breast cancer patients are premenopausal, with the average age reported as 50 to 53 years in various studies [4].

In Mexico, cervical cancer remains a significant health care problem in many areas of the country. Breast cancer, on the other hand, is being detected at higher rates, and, in some parts of the country, it has surpassed cervical cancer as the number one cancer in women. The most recent registry data report around 12,500 breast cancer cases with around 4000 deaths from the disease. Although breast cancer seems to be diagnosed at a younger age than in more developed countries (more than a decade younger at diagnosis), this seems to be partially related to the number of younger people in Mexico [7].

In Africa, the trends are difficult to evaluate; however, local registries report a doubling of rates over the past 40 years, but the degree to which these figures represent real increases, as opposed to changes in disease tracking and reporting, is unclear. 

In the Arab world, a scarcity of data is remarkable; some countries either have a national or regional registry, while others have no data. However, breast cancer is the most common cancer in Arabic women and affects younger women than their counterparts in industrialized nations. In Lebanon, the age-standardized incidence rates were 46.7/100 000 in 1998, 69/100 000 in 2004, and 84/100 000 in 2007. Although these figures remain lower than that observed in developed countries, they are substantially higher than in other developing countries of the region or the non-Jews in Israel. This is probably attributed to the wide adoption of screening programs and to a better awareness of breast cancer and its early signs. Clinical observation from other Arab countries and comparative age-standardized rates and age-specific standardized incidences show that breast cancer is seen in younger groups. The ASR for breast cancer in women younger than 50y in Lebanon is 153/100 000 in 1998, 194/100 000 in 2004, and 286/100 000 in 2007, compared to 42.5/100,000 for breast cancer cases in the United States. The median age of breast cancer in Lebanon is 52 years, with 49.1% of cases younger than 50, compared to 63 years for developed countries such as the US with 50% of cases over 65 years of age [1, 8, 9]. 

The Middle East Cancer Consortium (MECC) presents information about cancer incidence for populations in Cyprus, Egypt (Gharbia Region), Israel (Jews and Arabs), and Jordan for the period 1996–2001. The MECC findings are compared with those from the US Surveillance, Epidemiology, and End Results (SEER) Program: age-standardized incidence rates (ASRs) per 100,000 females were highest among Israeli Jews (93.1), similar to the rates reported in the US SEER females (97.2). These rates were significantly higher than those reported in Cypriot (57.7), Egyptian (49.6), Jordanian (38.0), and Israeli Arab (36.7) females. The high incidence rates described in Israeli Jews were similar to those described in North American and West European countries, while the lower rates in the other Middle Eastern groups were more similar to rates in Mediterranean Europe, Eastern Europe, and some of Asia and Africa [10].

Comparisons with SEER data reveal that Pakistan has a younger patient population with larger tumors, higher grades, and more advanced disease stage on presentation than does the United States. Receptor-negative tumors are also more common [11].

## 3. Screening and Early Detection

Proper screening necessitates the presence of certain elements which include high-quality screening using mammography, high coverage and participation and effective referral systems for diagnosis and treatment [12]. It is costly to implement such screening strategies thereby rendering screening unfeasible in low- and middle-income countries (LMCs). It is necessary that screening be implemented in LMCs in order to facilitate early detection and avoid advanced stage cancers at presentation. However, with mammography being the gold standard of screening in developed countries, it is arguable whether to put it into use in LMCs. Major obstacles involve high cost, lack of health care infrastructure, encouraging women to get screened, and high incidence of breast cancer occurring in younger populations of women aged 40–49 in which no significant benefit of mammography was seen in this age group [4, 13, 14]. For these reasons of impracticality, the Breast Health Global Initiative (BHGI) was founded in order to develop feasible, cost-effective, and culturally specific guidelines for LMCs. The guidelines were constituted of four levels of resources which include basic, limited, enhanced, and maximal. The basic-level resources were constituted of basic breast health awareness through education and self-examination and a clinical breast exam (CBE). The limited level includes CBE in addition to diagnostic ultrasound with or without mammography within limited financial means and modest health care systems. The third level involves mammography screening and the maximal level includes population-based mammographic screening [15]. Using this stepwise-based approach, advancement in LMCs health care systems may be achieved. 

There is emerging evidence that awareness campaigns in LMCs can improve adherence to screening guidelines. In Lebanon, four national surveys were conducted in collaboration with the National Breast Cancer Awareness Campaigns. These surveys revealed a slight improvement in utilization of mammography from 11% to 18% within a period of 12 months [16]. These results reveal an increased tendency to use mammography, however, at a low pace. Guidelines for screening of breast cancer have been established in Lebanon. These include a mammography scan every year starting at the age of 40, women with family history of breast cancer should start screening 10 years prior to the onset of the first case in the family, all women are to have an annual CBE with mammography and one CBE every three years between the age of 20 and 40, all women are to perform breast self-examination (BSE) once per month starting at the age of 20, two routine views are needed for a valid mammography which include craniocaudal and lateral oblique, and finally ultrasound is not recommended for asymptomatic women [17].

## 4. Clinical Presentation and Pathology

Although <5% of breast cancer diagnosed in mammographically screened population are stage III tumors [18], the reality is different from that in LMCs. With inadequate resources for early detection of breast cancer, the majority of patients from LMCs presents with advanced or metastatic disease. In a recent study done in Pakistan, the clinical presentation of patients was highest in advanced stages III and IV amounting to 65.7% in poor societies and 43.6% in middle- and high-income societies [11]. Both of these values are significantly higher than the 18.9% and 11.3% incidence of stage III and IV among African-Americans and American Whites, respectively, [18]. In another study in Nigeria, 72% of patients presented with stage III or IV disease [5]. In Arab countries, the data also show similar trends with a rate of advanced or metastatic stage reaching 60 to 80% of cases [1]. In Egypt, stages III and IV constitute 68% of all breast cancer cases [1, 19, 20]. As for Saudi Arabia, stages III and IV constitute about 46% of cases [21]. Also in Oman the rates are high amounting to 50.8% [22]. Efforts aimed at early detection can decrease stage at diagnosis and potentially improve the probability of survival and cure. 

Histologic diagnosis is necessary before initiating treatment. Choosing the type of biopsy whether fine needle aspiration cytology, core needle biopsy, or excisional biopsy depends on the resources available. Again the BHGI has defined the basic level of diagnosis and pathology to include history, CBE, physical exam, and interpreting specimens from surgical biopsy and fine needle aspiration to include tumor size, lymph node status, histologic type, and tumor grade. The limited level includes mammographic or ultrasound imaging of the breast. Core needle biopsy and image-guided sampling are used in order to determine estrogen receptor and progesterone receptor status as well as margin status. Also, within this level, resources should be allocated to include evaluation for metastasis with chest X-rays, ultrasound of liver, as well as blood studies. As for the enhanced level of diagnosis, it includes preoperative needle localization under mammographic or ultrasound guidance, and improved services involve the presence of a cytopathologist. Higher-level resources within this level could include bone scanning for metastatic examination. Finally, the maximal level could involve stereotactic biopsy, HER-2/neu status, sentinel node biopsy, immunohistochemistry staining of sentinel nodes for cytokeratin to detect micrometastases, and several imaging studies that include the use of CT scanning, PET scanning, MIBI scanning, as well as breast MRI [15, 23].

## 5. Adequacy of Management

 Mastectomy rates in Arab countries are high amounting to 79.9%–82% in Egypt, 65% in Oman, 70% among Palestinians, 88% in Syria, and 82.4% in Tunisia [1, 20, 22, 24–26]. In other LMCs, the picture is very similar. In India, the majority of patients present with stage IIIa and IIIb tumors reaching levels of 62%. Modified radical mastectomy after or without neoadjuvant chemotherapy is the most commonly used procedure for almost all types of breast cancers [27].

A major reason behind this practice could be related to the advanced stage that patients present with and the low number of radiation centers since radiation therapy is important in the treatment of breast cancer after breast conserving surgery [5, 28]. This necessitates the presence of sufficient radiation facilities and establishing early detection methods in order to provide proper treatment upon detection with higher survival rates of less-advanced disease stages. 

The younger age at presentation in LMCs has been associated with a worse outcome compared to patients presenting at an older age. In a study from Lebanon, women younger than 35 years at presentation had a worse survival despite receiving more chemotherapy and adequate hormonal therapy compared to women presenting with the disease between 35 and 50 years and women older than 50 years old [28].

## 6. Treatment Guidelines

The BHGI has also defined treatment guidelines for each stage at presentation according to the resource level available. In stage I breast cancer, the primary treatment modality at the basic level constitutes modified radical mastectomy due to the unavailability of radiation therapy. Systemic therapy at this level includes the incorporation of tamoxifen in both premenopausal and postmenopausal women. At the limited level, breast-conserving surgery as well as sentinel lymph node biopsy with blue dye becomes feasible, together with the incorporation of adjuvant anthracycline-based chemotherapy such as adriamycin, cyclophosphamide, or classical CMF (cyclophosphamide, methotrexate, and 5-FU). Moving to the enhanced level of resources, sentinel lymph node biopsy using radiotracer, breast-reconstruction surgery, as well as breast-conserving whole-breast irradiation as part of breast conserving surgery become applicable. Adjuvant chemotherapy may include the use of taxanes, while adjuvant endocrinal therapy may include the use of aromatase inhibitors and luteinizing hormone-releasing hormone (LHRH) agonists. Within this level as well, use of trastuzumab for HER2-neu-positive disease becomes feasible. At the maximal level, dose-dense chemotherapy can be implemented. In stage II, the basic level includes the use of cytotoxic chemotherapy in addition to modified radical mastectomy and systemic hormonal therapy that are the basic treatment strategies for stage I breast cancer at this level. Another difference between stage I and II breast cancer includes the incorporation of postmastectomy irradiation of chest wall and regional nodes for high-risk cases at the limited level [29]. Locally advanced breast cancer (LABC) represents a wide variety of clinical presentations including large tumors (>5 cm), extensive regional lymph node involvement, direct involvement of the underlying chest wall or skin with edema or satellite skin nodules together with inflammatory breast cancer. In the TNM staging classification of LABC is represented by stages IIIA, IIIB, and IIIC [30]. In countries of limited resources, LABC is considered to be the commonest form of presentation as well [31, 32]. Combined modality therapy utilizing neoadjuvant chemotherapy followed by locoregional therapy (surgery, radiation, or both) is emerging globally as the standard of care for LABC including inflammatory breast cancer. At the basic level of resources, patients are treated with neoadjuvant or adjuvant anthracycline-based chemotherapy and modified radical mastectomy. Preoperative or postoperative hormonal therapy may be given to patients with medical comorbidities who are unable to tolerate systemic chemotherapy. Postmastectomy irradiation of chest wall and regional lymph nodes becomes incorporated at the limited level of resources. Efforts should be made to incorporate it at the basic level as well. Breast-conserving surgery, breast-reconstruction surgery, and breast-conserving whole breast irradiation as part of breast conserving therapy are feasible by shifting to the enhanced and maximal levels. With neoadjuvant chemotherapy, a large number of patients are undergoing conservative surgery with axillary dissection and radiation [29]. However, for patients to undergo breast conservation, they require to meet certain criteria because patients with locally advanced disease are more likely to have locoregional recurrence. Criteria for patients undergoing conservative therapy after chemotherapy include a solitary primary tumor ≤4 cm in size or two primary tumors within a sphere <4 cm, absence of multiple breast calcifications, no skin involvement, the ratio of tumor to breast size is small enough for good cosmetic result, clinically node negative, absence of extensive involvement of breast or dermis, and no contraindications for radiotherapy [33]. Inclusion of taxanes, aromatase inhibitors, as well as trastuzumab (for HER2-positive disease) becomes implemented at these levels as well. 

Patients with metastatic breast cancer are unlikely to be cured of their disease by any means. Available therapeutic modalities are local (radiation therapy or surgery) or systemic therapy (endocrine therapy, chemotherapy, or molecularly targeted therapies), most of which are not present in countries with limited resources. Thus, at the basic level, locoregional treatment available is total mastectomy for ipsilateral breast tumor recurrence and the use of tamoxifen as systemic therapy. These resources extend to include palliative radiation therapy and anthracycline-based chemotherapy or CMF at the limited level. Sequential combination chemotherapy, use of trastuzumab, lapatinib, and bisphosphonates become available at the enhanced level. These resources in addition to bevacizumab and fulvestrant become incorporated into the management strategy at the maximal level of resources [29].
